# Clinico-radiological evaluation and management of type II radial ray defect in a young female from rural India: case report

**DOI:** 10.11604/pamj.2022.43.189.36312

**Published:** 2022-12-13

**Authors:** Anshul Sood, Rajasbala Pradeep Dhande, Vadlamudi Nagendra, Gaurav Mishra, Gopidi Nidhi Reddy

**Affiliations:** 1 Department of Radiodiagnosis, Datta Meghe Institute of Higher Education and Research, Sawangi, Wardha, Maharashtra, India

**Keywords:** Radial ray defect, radial dysplasia, radial ray anomaly, case report

## Abstract

Radial Ray Defect (RRD) is a rare disorder, and the etiology of this disorder is still under discussion. RRD is associated with many medical conditions for which prenatal counselling is of paramount importance. Any association with the family history is still unknown. The patient is a 16-year-old female who came to the orthopaedic clinic complaining of tingling and weakness in the right forearm. On examination, there was a gross deformity in the right forearm with radial deviation of the hand. An X-ray revealed radial ray defect type-2. She previously had a history of perinatal infection during early childhood. Surgery was successfully achieved, and positive results were accomplished. Radial Ray Defect can be focal or associated with other clinical manifestations. The timing of antenatal ultrasound for detecting this musculoskeletal anomaly is crucial. When RRD is associated with other syndromes, counselling to the parents about the quality of life and morbidity comes into play. Treatment is primarily surgical.

## Introduction

Radial dysplasia is significant when the structures on the radial side of the forearm (radius, carpals on the radial side and the thumb) fail to develop normally [1]. It is rare, with an incidence of 1 in 30,000 live births [2]. Radial ray syndrome is associated with hypoplasia or aplasia of the radius with/without deficient thumb and carpal bones. Radial ray syndrome and associated malformations range from unilateral to bilateral involvement. It can also have sporadic occurrences associated with multiple malformation syndromes. Isolated RRD comprises 8% to 30% of the cases, and the majority of the cases are associated with other anomalies [3]. The aim of this case study was to report our experience with a young female from rural India with radial ray anomaly and its management.

## Patient and observation

**Patient information:** a 16-year-old female child came to the orthopaedic clinic complaining of weakness and tingling sensation in the right forearm since one year. The patient´s mother noticed a deformity in the right forearm in the perinatal period. The patient had a history of perinatal infection of the right forearm associated with discharge at the age of 6 weeks which continued till five months. The patient was managed by surgery (documentation not available) and below the elbow brace, following which she was advised to follow up at 18 years. The patient´s mother had non-consanguineous marriage and had not undergone any antenatal ultrasound scans due to her low socio-economic status. There was no significant family history. Patient has no comorbidities.

**Clinical findings:** on inspection, there was gross deformity with the prominence of the right ulnar head and the presence of a non-tender scar on the dorsolateral aspect of the right ulna. On physical examination of the right hand, done in a supine and sitting position, dorsiflexion was limited to 0-10°, palmar flexion was limited to 0-10° with valgus deformity of 0-70° and radial deviation of 70-90°. The supinator muscle was absent, and the forearm remained in pronation on rest. The patient was unable to make a fist and unable to flex the thumb.

**Timeline of current episode:** January 2021: patient referral to the department of Radiology. X-ray and CT were conducted. March 2021: surgical correction was performed. October 2021: follow up.

**Figure 1:**
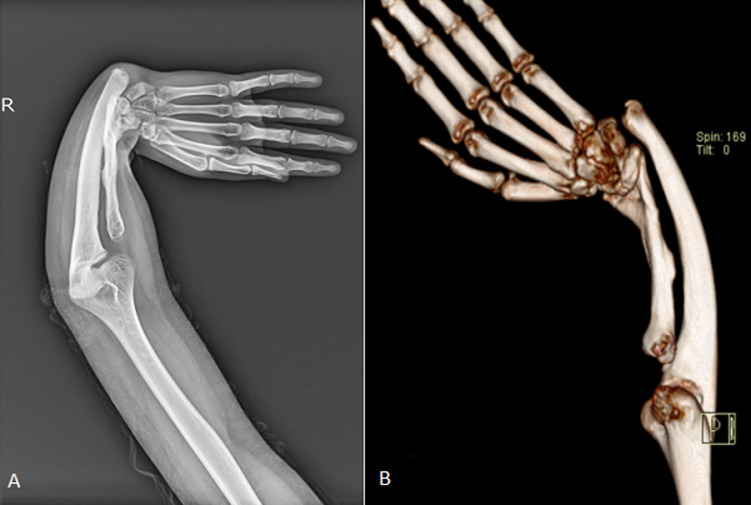
X-ray antero-posterior view (A) and computed tomography 3D reconstruction (B) of right forearm showing grossly shortened radius, deformed radius and ulna with radio ulnar dislocation

**Figure 2:**
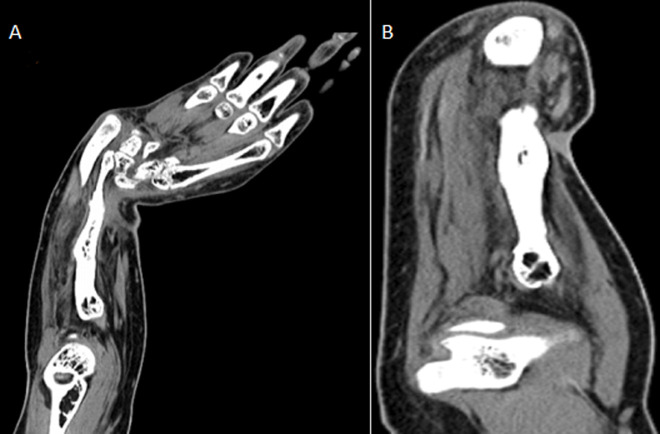
computed tomography coronal (A) and sagittal (B) view of right forearm showing non-visualization of the proximal 1/3^rd^ of the radius with the shaft of ulna appearing curved and poorly supporting the wrist joint; muscles of the forearm show disuse atrophy

**Diagnostic assessment:** X-ray imaging of the right forearm revealed a grossly shortened radius, deformed radius and ulna and radio ulnar dislocation, as shown in Figure 1. C.T. scan revealed non-visualization of the proximal 1/3rd of the right radius, with the shaft of the ulna appearing curved and poorly supporting the wrist joint. Muscles of the right forearm appeared atrophied, as shown in Figure 2. On blood examination, TSH was raised, haemoglobin levels were low (11.1 mg/dl) with a peripheral smear showing normocytic hypochromic R.B.C.s with mild anisopoikilocytosis, few microcytes and pencil cells.

**Diagnosis:** based on clinical and radiological examination, a diagnosis of radial ray defect type-2 was made. Deformity correction via operative procedure was advised after one week.

**Figure 3:**
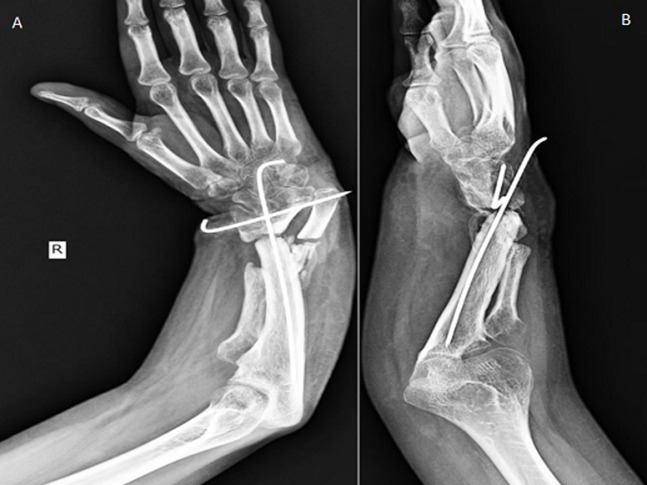
post-operative (osteotomy of radius and ulna) X-ray antero-posterior (A) and lateral (B) view of right forearm showing reduction of the deformity using a fibular bone graft and k-wire stabilization

**Therapeutic intervention:** after 1-week, a surgical fitness procedure of the patient was performed, and the following day surgical intervention was done in which deformity of the right radius and ulna was corrected by osteotomy. Alignment of the proximal ulna with distal radius with K-wire and bone grafting was performed under general anaesthesia and a brachial plexus nerve block, as shown in Figure 3. Patient was discharged after 2 weeks with post-operative cast for 6 months.

**Follow-up and outcome:** postoperative X-ray revealed two K wires with bone graft in situ in the right forearm. On discharge, the patient was advised physiotherapy, limb elevation and hand exercises. On routine follow-up after 6 months, the patient is able to perform her day-to-day activities with minimal difficulties.

**Patient´s perspective:** translated from the patient´s mother´s own language. There was some noticeable defect in the right forearm of my child since birth. Later my child suffered a right forearm infection associated with discharge at the age of 6 weeks, for which surgery was done (documents not available). Post-surgery doctor advised a below elbow brace and asked to come back at 18 years of age to correct the deformity. At present, at the age of 16 years, she was having tingling sensation, pain and decreased movements in the right forearm. So, we came for further treatment. The doctor advised an X-ray and said that there was a defect in the right forearm bone and advised an operation. After successful surgery, I am happy to see my child do her day-to-day activities with minimal difficulties.

**Informed consent:** informed and written consent was obtained from the patient.

## Discussion

Focal musculoskeletal anomalies can be isolated or can be in association with clinical syndromes or can be affected by Central Nervous System (CNS) anomalies, genetic disorders, skeletal anomalies, and/or karyotype abnormalities [4]. Radial dysplasia is significant when the structures on the radial side of the forearm (radius, carpals of radial side and the thumb) fail to develop normally [1]. Radial ray syndrome is associated with hypoplasia or aplasia of the radius with/without deficient thumb and carpal bones.

The distal end of the limb bud in the embryo has an Apical Ectodermal Ridge (AER) which covers proliferating mesenchyme known as the progress zone. The posterior border of this progress zone is known as the Zone of Polarising Activity (ZPA). Fibroblast Growth Factor type 4 (FGF 4) regulates the AER, which in turn controls limb growth in a proximal to distal fashion. However, the Sonic hedgehog (Shh) gene regulates the ZPA, which is responsible for limb patterning along the anterior to posterior axis [5]. The underlying cause of RRD is still unknown. However, signals from the zone of polarising activity in the vertebrate limb bud are known to control the pattern of cellular differentiation in a radioulnar direction [6,7].

Bayne and Klug classified Radial Ray Disease, which includes four types based on progressive radiographic severity of the deficiency of the radius. It adequately defines type 1 (deficient distal radial epiphysis), type 2 (deficient distal and proximal radial epiphyses), type 3 (partial radius dysplasia), and type 4 (complete absence of the radius) [8]. Limbs develop by the end of 12 weeks. Hence should be visualised in the antenatal ultrasound scan scheduled for nuchal translucency (NT) scan at 11-13 weeks to detect any abnormality in the musculoskeletal system. This helps detect any association with the following syndromes, which may require prenatal genetic counselling such as: 1) Holt-Oram syndrome is associated with cardiac defects and congenital defects of the thumb; 2) Thrombocytopenia Absent Radius (TAR), which is autosomal recessive, comprises thrombocytopenia and absent radius; 3) Vertebral, Anal, Cardiac, Tracheoesophageal, Esophageal, Renal et Limb (VACTERL) association consists of vertebral, anal, and cardiac anomalies, trachea-oesophagal fistula, and radial and limb defects; 4) Chromosomal anomalies like trisomy 13 and trisomy 18; 5) Fanconi anaemia may be associated with hydrocephaly, abnormally shaped ears, kidney problems, and abnormal thumb and forearm.

Fetal blood sampling is always advised to rule out the possibility of TAR syndrome or Fanconi´s anaemia [9]. Management is surgical, but in infants, the management is conservative with the limited role of splint; hence initially, wrist and elbow range of movements with improving the radial and flexion deformities by stretching exercises is considered. To stretch the stiff structures, serial casts are used on the radial side to achieve longitudinal alignment, but the carpal malalignment cannot be corrected. The operative procedures include radialisation, in which the head of the ulna is brought under the radial carpal bones, and the hand is fixed in ulnar deviation with K wire. Another operative procedure is centralisation which attempts to re-align the 3^rd^ metacarpal at the right angles to the plane of the distal ulnar epiphyseal plate is done.

## Conclusion

Radial ray is a rare disorder associated with radial displacement of the phalanges and/or the carpal and metacarpal bones due to improper support to these bones with underdeveloped muscles on the radial side. It may present as an isolated defect or may be associated with other syndromes; hence, prenatal detection of this anomaly is of paramount importance. Counselling should be given to the parents about the postnatal complications and prognosis of the disorder. Postnatal X-ray is considered the best diagnostic technique. Correcting this deformity is best done by surgical approach, keeping centralization as its primary intention.
